# Supplementary material for: Prevalence and determinants of depression, anxiety, and stress among the elderly population in Bangladesh: A cross-sectional study
Source: PLoS One. 2026 Mar 20;21(3):e0345505. doi: 10.1371/journal.pone.0345505 (PMC13004408; doi:10.1371/journal.pone.0345505)
Supplement: S1 Table — (DOCX) [file pone.0345505.s001.docx]

**Table S1:** Frequency n (%) of aged 60 years and older by socioeconomic characteristics in Bangladesh

| **Characteristics** | | **Frequency** |
| --- | --- | --- |
| Sex | Male | 363 (90.75) |
|  | Female | 37 (9.25) |
| Religion | Islam | 376 (94.0) |
|  | Hindu | 24 (6.0) |
| Region | Rural | 189 (47.25) |
|  | Urban | 211 (52.75) |
| District | Dhaka | 124 (31.0) |
|  | Nilphamari | 74 (18.5) |
|  | Mymensingh | 103 (25.75) |
|  | Satkhira | 99 (24.75) |
| Educational status | No-qualified education | 178 (44.5) |
|  | Secondary | 86 (21.5) |
|  | Higher Secondary | 48 (12.0) |
|  | Graduate | 46 (11.5) |
|  | Postgraduate | 42 (10.5) |
| Retirement status | Yes | 211 (52.75) |
|  | No | 189 (47.25) |
| Physical status | Good | 88 (22.0) |
|  | Average | 197 (49.25) |
|  | Bad | 100 (25.0) |
|  | Very bad | 15 (3.75) |
| Living status | Joint family | 202 (50.5) |
|  | Single family | 197 (49.25) |
|  | Old age home | 1 (0.25) |
| Communication with children and grandchildren | Often | 273 (68.25) |
|  | Sometimes | 98 (24.5) |
|  | Less | 18 (4.5) |
|  | None | 11 (2.75) |
| Receive Medical and other support from family | Good | 232 (58.0) |
|  | Fairly | 129 (32.25) |
|  | None | 39 (9.75) |
| Financial support to the family | Yes | 255 (63.7) |
|  | No | 145 (36.3) |
| Relationship with family as get older | Good | 273 (68.25) |
|  | Fairly | 103 (25.75) |
|  | None | 24 (6.0) |
| Opinion in the family | Valuable | 275 (68.75) |
|  | Less | 99 (24.75) |
|  | None | 26 (6.5) |
| Involvement in social activities | Yes | 159 (39.75) |
|  | No | 241 (60.25) |
| Communication with neighbors | Often | 223 (55.8) |
|  | Sometimes | 138 (34.5) |
|  | Less | 29 (7.2) |
|  | Very Less | 10 (2.5) |
| Encountered any discrimination or prejudice related to age in society | Yes | 98 (24.5) |
|  | No | 302 (75.5) |
| Experienced social isolation and loneliness | Yes | 157 (39.25) |
|  | No | 243 (60.75) |
| Difficulty accessing transportation, public places or services due to age | Yes | 165 (41.25) |
|  | No | 235 (58.75) |
| Experienced changes in social relationships or support since turning 60 | Yes | 183 (45.75) |
|  | No | 217 (54.25) |
| Social status after the age of 60 | Good | 191 (47.75) |
|  | Fairly | 176 (44.0) |
|  | Bad | 33 (8.25) |
