# Supplementary material for: Prevalence and determinants of depression, anxiety, and stress among the elderly population in Bangladesh: A cross-sectional study
Source: PLoS One. 2026 Mar 20;21(3):e0345505. doi: 10.1371/journal.pone.0345505 (PMC13004408; doi:10.1371/journal.pone.0345505)
Supplement: S2 Table — (DOCX) [file pone.0345505.s002.docx]

**Table S2:** Test of Reliability and Validity

| **Constructs** | **Items** | **Loading** | **CR** | **Cronbach’s Alpha** |
| --- | --- | --- | --- | --- |
| Depression | DeS2 | 0.63 | 0.692 | 0.701 |
|  | Des4 | 0.59 |  |  |
|  | DeS6 | 0.5 |  |  |
|  | DeS8 | 0.52 |  |  |
|  | DeS9 | 0.54 |  |  |
| Stress | SS1 | 0.52 | 0.716 | 0.715 |
|  | SS2 | 0.64 |  |  |
|  | SS3 | 0.56 |  |  |
|  | SS6 | 0.53 |  |  |
|  | SS10 | 0.64 |  |  |
| Anxiety | AS1 | 0.69 | 0.771 | 0.770 |
|  | As2 | 0.65 |  |  |
|  | AS3 | 0.64 |  |  |
|  | AS4 | 0.54 |  |  |
|  | AS5 | 0.54 |  |  |
|  | AS7 | 0.53 |  |  |
| Socio-Demographic | SD7 | 0.53 | 0.775 | 0.774 |
|  | SD13 | 0.51 |  |  |
|  | SD14 | 0.64 |  |  |
|  | SD15 | 0.72 |  |  |
|  | SD18 | 0.53 |  |  |
|  | SD19 | 0.55 |  |  |
|  | SD22 | 0.53 |  |  |
