# Supplementary material for: Prevalence and determinants of depression, anxiety, and stress among the elderly population in Bangladesh: A cross-sectional study
Source: PLoS One. 2026 Mar 20;21(3):e0345505. doi: 10.1371/journal.pone.0345505 (PMC13004408; doi:10.1371/journal.pone.0345505)
Supplement: Attachment — Submitted filename: Point-by-point response to reviewers.pdf [file pone.0345505.s005.pdf]

Authors' responses to the review comments:

We would like to sincerely thank the anonymous reviewers and the Academic Editor for their valuable comments. We have considered all comments and then thoroughly revised and formatted the manuscript. A detailed response to each comment is provided in the tables as follows.

**Editor comments:**	**Response**	**Note**
PONE-D-25-55520<br>Prevalence and Determinants of Depression, Anxiety, and Stress Among the Elderly Population in Bangladesh: A Cross-sectional Study<br>PLOS ONE<br>Dear Dr. Moyazzem,<br>Thank you for submitting your manuscript to PLOS ONE. After careful consideration, we feel that it has merit but does not fully meet PLOS ONE's publication criteria as it currently stands. Therefore, we invite you to submit a revised version of the manuscript that addresses the points raised during the review process.<br>Please submit your revised manuscript by Jan 11 2026 11:59PM. If you will need more time than this to complete your revisions, please reply to this message or contact the journal office at plosone@plos.org. When you're ready to submit your revision, log on to https://www.editorialmanager.com/pone/ and select the 'Submissions Needing Revision' folder to locate your manuscript file.<br><br>Please include the following items when submitting your revised manuscript:<br>A rebuttal letter that responds to each point raised by the academic editor and reviewer(s). You should upload this letter as a separate file labeled 'Response to Reviewers'.<br>A marked-up copy of your manuscript that highlights changes made to the original version. You should upload this as a separate file labeled 'Revised Manuscript with Track Changes'.<br>An unmarked version of your revised paper without tracked changes. You should upload this as a separate file labeled 'Manuscript'.<br>If you would like to make changes to your financial disclosure, please include your updated statement in your cover letter. Guidelines for resubmitting your figure files are available below the reviewer comments at the end of this letter.	Thanks. We appreciate your feedback. As per comments, a careful revision has been conducted, and all required files are uploaded to the journal submission system.	The revised texts are highlighted in "red" color.
**Journal Requirements:**	**Response**	**Note**
When submitting your revision, we need you to address these additional requirements.<br>1. Please ensure that your manuscript meets PLOS ONE's style requirements, including those for file naming. The PLOS ONE style templates can be found at<br>https://journals.plos.org/plosone/s/file?id=wjVg/PLOS	Thank you very much, we revised the manuscript following the PLOS ONE style.	The revised texts are in "red". Page: 18-23

One_formatting_sample_main_body.pdf and https://journals.plos.org/plosone/s/file?id=ba62/PLOSOne_formatting_sample_title_authors_affiliations.pdf		
2. In the online submission form you indicate that your data is not available for proprietary reasons and have provided a contact point for accessing this data. Please note that your current contact point is a co-author on this manuscript. According to our Data Policy, the contact point must not be an author on the manuscript and must be an institutional contact, ideally not an individual. Please revise your data statement to a non-author institutional point of contact, such as a data access or ethics committee, and send this to us via return email. Please also include contact information for the third party organization, and please include the full citation of where the data can be found.	Thanks. We revised the data available statement. We also submit the data file as a supplementary file.	Page: 23
3. Your ethics statement should only appear in the Methods section of your manuscript. If your ethics statement is written in any section besides the Methods, please move it to the Methods section and delete it from any other section. Please ensure that your ethics statement is included in your manuscript, as the ethics statement entered into the online submission form will not be published alongside your manuscript.	Thank you very much for your insightful comments and feedback. We added the ethics statement in the Methodology section.	Page: 5
**Reviewer #1 Comments**	**Response**	**Note**
The manuscript is generally well written. The authors would benefit by incorporating the following articles in the introduction and discussion sections-<br>https://doi.org/10.1007/978-981-99-9153-2_11<br>https://doi.org/10.9790/0837-2108025873	Thank you very much for your insightful comments and feedback. We believe that it helps to enhance the quality of the manuscript. We revised the Introduction section and cited the suggested papers.	The revised texts are in “red”. Page: 2
In the discussion, the authors acknowledge their results differ from some previous studies but don't deeply explore why these differences exist beyond general contextual factors. The manuscript could have benefited from more discussion about the cultural context of mental health in Bangladesh. Most importantly, study identifies important risk factors like social isolation and poor health status, it doesn't adequately address the potential confounding variables or explore the complex interplay between multiple risk factors.	Thanks. The main reason for the different results may be sampling variability. We added it to the Discussion section and the Limitations section.	Page: 22
**Reviewer # 2 Comments**	**Response**	**Note**
Thank you for the opportunity to review this manuscript, which examines the important topic of mental health among the elderly in Bangladesh. The use of validated tools (PHQ-9, GAD-7, PSS-10) and the attempt to use advanced statistical methods (SEM) are noted strengths.<br>However, I must express serious concerns regarding the rigor, preparation, and reporting of this study. I encountered several major errors in the text that appear to be careless copy-paste remnants from other	Thank you very much for your comments and feedback. We believe that it helps to improve the quality of the manuscript.<br><br>We revised the manuscript accordingly.	Page: 4

documents. These errors severely undermine my confidence in the carefulness of the entire work. Furthermore, the methodology lacks critical details necessary to verify the claim of "random" sampling, and the statistical reporting needs significant improvement.<br>I cannot recommend this manuscript for publication in its current state. It requires a major overhaul to address these fundamental issues.		
Specific Major Concerns:<br>1. I was alarmed to find text in the Results section that has absolutely nothing to do with this study. On page 4, under the 'Results' heading, the very first paragraph ends with: "The prevalence of stunted, underweight, and wasted children and their composition are presented in Figure 1." This is a study about the elderly, not malnourished children. The presence of such a glaring error suggests that the manuscript was not thoroughly proofread before submission. Furthermore, in the Discussion (Page 18), there is a placeholder left in the text: "...revealed the prevalence to be 11.4%, 10.0%, and 1.4%, respectively, by [NO_PRINTED_FORM] Conversely..." These errors are unprofessional and must be corrected. They also lead me to question if other parts of the data or text were inadvertently carried over from different projects.	Thanks. We revised the Results section.	Page:6, 22
2. The authors state: "total of 400 elderly population aged 60 and above were selected using stratified random samples from the Dhaka, Nilphamari, Mymensingh, and Khulna districts." This is a very strong claim that is currently unsupported by the text. To conduct true stratified random sampling, you would need a complete sampling frame (a list of every single elderly person in these four districts) from which to randomly draw participants. Did such a list exist? If you did not use a master list, this is likely not random sampling, but rather convenience or purposive sampling (perhaps with quotas for each district). You must honestly describe exactly how a participant was recruited. Did you go door-to-door? Did you visit clinics? "Random" has a specific statistical meaning; if standard randomization procedures weren't followed, you must change this terminology and list it as a major limitation, as it affects the generalizability of your prevalence findings.	Thanks. We revised the Methods section.	Page:4.
3. The sample size calculation needs clarification. You state: "According to p=0.05, CI=95% (Confidence Interval), d=0.05, and a power of 80%, the estimated sample size was 383 participants (16)." Usually, in prevalence studies, 'p' stands for the expected prevalence. Did you really expect only a 5% prevalence (p=0.05) of mental health issues? If the expected prevalence is unknown, it is standard practice to use p=0.5 (50%) to yield the maximum sample size for a	Thank you very much. We revised the Methods section.	Page:4.

given precision. If you used p=0.05 to get N=383, your study might be underpowered if the actual prevalence is much higher (which your results show it is). Please clarify this calculation.		
4(a). You have used ordinal logistic regression, which is appropriate for your ranked outcome data. However, this model assumes the "proportional odds" assumption (i.e., the relationship between predictors and the outcome is the same across all cut-points of the severity scale). You must test this assumption and report whether it was met. If it was violated, a different model (like multinomial logistic regression) might be needed.	Thanks. We revised the Methods section.	page: 5.
4(b). Some of your Confidence Intervals in Table 3 are very wide (e.g., Communication with children 'Often': OR 4.332, CI [1.127, 16.650]). This indicates low precision, likely due to small cell counts in those specific categories. You should acknowledge this lack of precision in your interpretation.	Thanks. We agreed with you. We mentioned it in the limitation section. This may be happed due to small sample of that category.	page:23
5. While SEM is a powerful tool, it is not entirely clear what unique value it adds here beyond the regression analysis, other than confirming that Depression, Anxiety, and Stress are correlated (which is already well-known). The hypotheses tested (H1-H5) are very basic. Please provide a stronger rationale in the introduction or methods for why SEM was necessary to answer your specific research questions.	Thanks. We revised the Methods section.	page: 5
Minor (but important) Concerns:<br>1. The abstract mentions "unknown risk factors" in the background. Many risk factors for elderly depression are actually quite well known globally. It would be better to frame this as a need to understand these factors specifically within the Bangladeshi context.	Thank you very much. We revised the Abstract of the manuscript.	page:1
2. The manuscript needs careful proofreading. Many sentences are grammatically incorrect or poorly phrased, making them difficult to understand.<br>(a) Example (Introduction): "This study aimed to find the people who were contributing to the financial, family, society, and world, and how they were spending their lives with mental health." This sentence is convoluted and its meaning is unclear.	Thank you. We checked the entire manuscript and corrected grammatical issues and typos.	page:1-23
b) Example (Conclusion): "Older people (aged 60 years and above) were in depression, anxiety, and stress in the end stage of life." The phrasing "were in depression" is awkward, and "end stage of life" is a very strong and likely inaccurate generalization.	Thanks. We revised the Conclusion section.	page:23.
(c) Example (Conclusion): "The harmful factors causing mental health problems in older people need to be diagnosed." Factors are identified or addressed, not diagnosed.	Thanks. We revised the Conclusion section.	page:23.
(d) Repetitive Text: The manuscript repeatedly uses the same awkward phrasing. For example, in Table 2's description, the same sentence about non-significant findings is repeated three times in a single paragraph.	Thanks. We revised the manuscript accordingly.	page:11, 14.
**Reviewer #3 Comments**	**Response**	**Note**

The article entitled "Prevalence and Determinants of Depression, Anxiety, and Stress Among the Elderly Population in Bangladesh: A Cross-sectional Study" employs a cross-sectional design that is methodologically consistent with the aims of the investigation. However, the gender distribution warrants attention: 363 of the 400 respondents are male, resulting in a markedly homogeneous sample. This imbalance introduces a potential sampling bias that limits the representativeness of the results and should be explicitly recognized as a methodological weakness.	Thank you very much for carefully checking the manuscript and providing comments and feedback. We believe that this helps to improve the quality of the manuscript.	The limitatio are mention ed in "Blue" page:23.
The ethical procedures described appear adequate; nonetheless, it should be noted that in several countries verbal consent is not considered sufficient, and the authors could provide more detail regarding how this process was conducted.	Thanks. We revised the ethical declaration.	Page: 4
The statistical analyses were performed appropriately, ensuring analytical rigor and supporting the validity of the reported findings. The results are coherent and provide empirical support for the study's conclusions.	Thanks for your positive comments. We appreciate it,	
With regard to the references, 27 of the 31 citations are more than five years old, which suggests that the manuscript would benefit from the inclusion of more recent literature to strengthen its theoretical and empirical foundation.	Thanks. We added some recent citations.	
Although the manuscript is understandable, the standard of English requires substantial revision. Several sections contain unclear or disconnected statements, such as:<br>• In the abstract, the results section includes the sentence "Significant risk factors include medical support from family, relationships with family as<br>• age progresses," which is confusing. A more precise formulation would be: "Significant risk factors include poor medical support from family and inadequate family relationships as age progresses."	Thank you very much for your careful checking. We revised the Abstract as per your suggestion.	Page: 2
• In the final paragraph of the introduction, the sentence "This study aimed to find the people who were contributing to the financial, family, society, and world, and how they were spending their lives with mental health" is not aligned with the surrounding text and lacks clarity.	Thanks. We revised the Introduction section.	page:3
• In the first paragraph of the Results section, the statement "The prevalence of stunted, underweight, and wasted children and their composition are presented in Figure 1" is inconsistent with the study's focus on the elderly population and appears to be an oversight.<br>In summary, despite the limitations observed, particularly regarding sampling composition and language clarity, the study contributes relevant findings to its field and addresses an important public health issue.	Thanks. We revised the results section.	Page:6

In conclusion, the revised version of the manuscript has been produced as per the review outcomes. So, we hope that you will be happy to see this greatly improved version. Once again, we would like to thank you all for your dedication, professional services, and cooperation.
